# Statistical methods for testing X chromosome variant associations: application to sex-specific characteristics of bipolar disorder

**DOI:** 10.1186/s13293-019-0272-4

**Published:** 2019-12-09

**Authors:** William A. Jons, Colin L. Colby, Susan L. McElroy, Mark A. Frye, Joanna M. Biernacka, Stacey J. Winham

**Affiliations:** 1 Department of Health Sciences Research, Mayo Clinic, Rochester, MN 55905 USA; 2 Lindner Center of HOPE, University of Cincinnati College of Medicine, Mason, OH 45040 USA; 3 Department of Psychiatry and Psychology, Mayo Clinic, Rochester, MN 55905 USA

**Keywords:** Bipolar disorder, X chromosome, Genetic association, Rapid cycling, Binge eating, Alcohol use disorder, Suicidality, X chromosome inactivation, Sex differences, X chromosome statistical analysis

## Abstract

**Background:**

Bipolar disorder (BD) affects both sexes, but important sex differences exist with respect to its symptoms and comorbidities. For example, rapid cycling (RC) is more prevalent in females, and alcohol use disorder (AUD) is more prevalent in males. We hypothesize that X chromosome variants may be associated with sex-specific characteristics of BD. Few studies have explored the role of the X chromosome in BD, which is complicated by X chromosome inactivation (XCI). This process achieves “dosage compensation” for many X chromosome genes by silencing one of the two copies in females, and most statistical methods either ignore that XCI occurs or falsely assume that one copy is inactivated at all loci. We introduce new statistical methods that do not make these assumptions.

**Methods:**

We investigated this hypothesis in 1001 BD patients from the Genetic Association Information Network (GAIN) and 957 BD patients from the Mayo Clinic Bipolar Disorder Biobank. We examined the association of over 14,000 X chromosome single nucleotide polymorphisms (SNPs) with sex-associated BD traits using two statistical approaches that account for whether a SNP may be undergoing or escaping XCI. In the “XCI-informed approach,” we fit a sex-adjusted logistic regression model assuming additive genetic effects where we coded the SNP either assuming one copy is expressed or two copies are expressed based on prior knowledge about which regions are inactivated. In the “XCI-robust approach,” we fit a logistic regression model with sex, SNP, and SNP-sex interaction effects that is flexible to whether the region is inactivated or escaping XCI.

**Results:**

Using the “XCI-informed approach,” which considers only the main effect of SNP and does not allow the SNP effect to differ by sex, no significant associations were identified for any of the phenotypes. Using the “XCI-robust approach,” intergenic SNP rs5932307 was associated with BD (*P* = 8.3 × 10^−8^), with a stronger effect in females (odds ratio in males (OR_M_) = 1.13, odds ratio in females for a change of two allele copies (OR_W2_) = 3.86).

**Conclusion:**

X chromosome association studies should employ methods which account for its unique biology. Future work is needed to validate the identified associations with BD, to formally assess the performance of both approaches under different true genetic architectures, and to apply these approaches to study sex differences in other conditions.

## Background

Although multiple genome-wide association studies have examined the genetic contributions to the risk of bipolar disorder (BD) [1, 2], few studies have examined the genetics of specific symptoms or comorbidities of BD. We previously identified several symptoms and comorbidities of BD that differ in prevalence by sex [3]. We found that rapid cycling (RC) and a lifetime history of a suicide attempt were more common for women than men and that men more frequently had a substance use disorder. Women are also more likely to have a comorbid eating disorder, particularly binge eating behavior (BE) [4]. The reason for these sex-specific differences in BD characteristics is unclear. However, many biological sex differences are thought to arise from either hormonal differences or from genetic differences (e.g., sex chromosomes). Brain development and function as well as psychiatric traits are influenced by sex hormone levels [5] and genetic factors [2]. For example, expression of the gene BDNF is influenced by estradiol, and a SNP within BDNF Val66Met has been shown to be associated with BD and other psychiatric traits [6]. The X chromosome contains many sex and reproductive genes influencing hormone levels, such as the androgen receptor (AR) [7]. Patients with X chromosome aneuploidies experience higher rates of various psychiatric disorders, including mood disorders [8]. Furthermore, X chromosome dosage and dosage compensation may be relevant for polygenic complex traits, such as BD [9].

Because males and females have different numbers of copies of the X chromosome, we hypothesize that X chromosome genetics might play a role in observed sex differences in BD. In particular, females carry two X chromosomes, while males carry only one, and the X chromosome in females (but not males) undergoes a process called X chromosome inactivation (XCI). This is an epigenetic process initiated by the long non-coding RNA *XIST* that triggers silencing of the inactive X, which results in males and females expressing similar levels of many X chromosome genes [10, 11]. The identity of the inactive X is random in humans [12], and the process is also tissue- and cell-specific [13, 14]. Furthermore, XCI does not affect all loci on the X chromosome. In fact, approximately 15% of X chromosome loci escape from XCI and are expressed from both X chromosomes in females [15], although these genes are not fully expressed from the inactive X. Escape genes include genes in the pseudoautosomal regions at the ends of the chromosome (PAR1 and PAR2), as well as gametologs (genes with homologous copies on X and Y, for which females have two copies on the X and males have one copy on X and one copy on the Y), and other genes escape variably [10]. The unique biology of the X chromosome means that applying approaches for analyzing autosomal genetic variants is not appropriate.

In this work, we develop a new approach for analyzing X chromosome genetic variants, which incorporates prior biological information on XCI status of various genes, and apply the approach to examine the role of X chromosome genetic variation in sex-specific symptoms of BD. Our approach combines existing approaches for testing marginal genetic associations within a logistic regression framework. We also consider a test that accounts for single nucleotide polymorphisms (SNP)-sex interactions to allow for different effects of X chromosome variants in males and females. We compare results across methods to enable assessment of potential strengths and limitations of each approach and report on our findings regarding the association of X chromosome variants with sex-specific symptoms and comorbidities of BD.

## Methods

In this study, we examined whether X chromosome variants are associated with sex-associated symptoms and comorbidities of BD. We utilized two cohorts of individuals with BD, one from the Mayo Clinic Bipolar Disorder Biobank [16] and one from the Genetic Association Information Network (GAIN) Study of BD [17], and we employed two different X chromosome-specific statistical approaches to assess associations between SNP genotypes and phenotypes. Rather than using a discovery-validation approach, a meta-analysis was conducted in order to boost sample size and reproducibility by combining the results derived from both cohorts (GAIN and Mayo).

### Mayo cohort

#### Subject selection

Individuals with BD (*N* = 969) from the Mayo Clinic Bipolar Disorder Biobank [16] (Mayo Bipolar Biobank) that had previously undergone genome-wide genotyping on the Illumina® Human OmniExpress BeadChip (Illumina®, Inc. San Diego, CA, USA) were included in this study. Control subjects (*N* = 777) that did not have BD or a psychiatric illness themselves or a first-degree relative with BD were selected from the Mayo Clinic Biobank [18]. This case/control set was previously analyzed [19] and was included in a large genome-wide association study conducted by the Psychiatric Genomics Consortium [2].

#### Phenotyping

Symptoms and comorbidities of BD were assessed through patient and clinical questionnaires [16]. Variables analyzed in this study included the symptom of rapid cycling (RC), comorbidities of binge eating behavior (BE), lifetime history of suicide attempt, and whether the individual had an alcohol use disorder (AUD), defined in the Diagnostic and Statistical Manual of Mental Disorders, 4th Edition (DSMIV) as a diagnosis of alcohol dependence or abuse [20]. Rapid cycling was defined as having four or more mood episodes within a year. Binge eating behavior was defined as an affirmative response to questions 5 and 6 of the Eating Disorders Diagnostic Scale [21]. These questions read “During the past 6 months have there been times when you felt you have eaten what other people would regard as an unusually large amount of food (e.g. a quart of ice cream) given the circumstances?” and “During the times when you ate an unusually large amount of food, did you experience a loss of control (feel you couldn’t stop eating or control what or how much you were eating)?” [21].

#### Genotyping

Quality control (QC) and imputation of genotyping data were performed using standard procedures as previously described [22]. Genetic ancestry was estimated with STRUCTURE [23, 24] using 1000 Genomes Project reference panels and used to exclude individuals of non-European ancestry. Genome-wide principal components were calculated to allow for adjustment for population substructure. X chromosome SNPs were imputed using IMPUTE 2.2.2 [25] with the 1000 Genomes Project reference panel (phase 1 data, all populations). Analyses were limited to X chromosome SNPs that had minor allele frequency above 0.05 and imputation *R*^2^ above 0.8. SNPs in the pseudoautosomal region (PAR) defined by GrCh37 were excluded due to low genotyping call rate.

### GAIN cohort

#### Subject selection

Cases with BD and controls without BD were recruited to the GAIN study and underwent phenotyping and genotyping as previously described [17] with data deposited in dbGaP [26] (accession number: phs000017.v3.p1). We used data from the subjects of European ancestry that passed genetic data QC (*N* = 1001 cases and *N* = 1034 controls).

#### Phenotyping

A history of BE, RC, suicide attempt, or an AUD was assessed in cases using the Diagnostic Interview of Genetic Studies (DIGS) (versions 2–4) [27]. Binge eating behavior was defined based on having affirmative responses to questions that addressed overeating and loss of control: “Has there ever been a time in your life when you went on food binges (i.e., rapid consumption of a large amount of food in a discrete period of time, usually less than two hours)?” and “During these binges were you afraid you could not stop eating, or that your eating was out of control?”. The presence of AUD was determined from the presence of any ICD 9 codes indicating DSMIII-R or DSMIV diagnoses of alcohol abuse (305.00; ICD-10 = F10.10) or alcohol dependence (303.90; ICD-10 = F10.20). Rapid cycling was defined as the presence of at least four mood episodes in a year.

#### Genotyping

Genotyping was performed using an Affymetrix™ Genome-Wide Human SNP Array 6.0 (Thermo Fisher Sientific, Inc., Waltham, MA, USA). Quality control was performed as previously described [17]. Imputation was performed as previously described [28]. SNPs analyzed were limited to those with MAF above 0.05 and imputation *R*^2^ above 0.8. SNPs in the PAR (defined by GrCh37) were excluded.

### Association testing

Because of the unique biology of the X chromosome, testing associations between X chromosome genetic variants and phenotypes requires different approaches than for autosomes. Previous work has used a logistic regression framework but coded the SNP variable differently depending upon the approach applied (Table 1). The coding approach historically implemented in the PLINK software [29] codes female genotypes as 0, 1, or 2 copies of the alternate allele and male genotypes as 0 or 1 copies of the alternate allele. This genotype coding ignores that XCI occurs and assumes that variants on both copies of the X chromosome are expressed in females (i.e., escape from XCI); this implicitly assumes that the effect of a change of a single allele has the same effect in females and males. As this is not true when a SNP is in a region that is inactivated, an alternate approach is to treat all SNPs as subject to XCI, using an approach originally proposed by Clayton [30]. Male genotypes are coded as 0 or 2 copies of the alternate allele, assuming that these male genotypes have the same effect as the respective homozygotes in females. Assuming that XCI is random across cells within a woman and random across women, female heterozygotes are viewed as an intermediate genotype, coded as 1. However, this also may not be optimal as 15% of X chromosome genes are expressed from both the active and inactive X chromosome. Given prior information regarding whether a region undergoes X chromosome inactivation, it is reasonable to consider this biological information when evaluating X chromosome associations.

**Table 1:** Different coding schemes for the SNP variable reflect different assumptions regarding XCI status

Assumed XCI status	Coding scheme	Coding of the SNP variable (Eq. 1)				Calculation of ORs^a^		
		Sex	# copies of effect allele					
			0	1	2	OR_M_	OR_W1_	OR_W2_
Subject	Clayton	M	0	*2*	NA	${e}^{2{\beta}_1}$	${e}^{\beta_1}$	${e}^{2{\beta}_1}$
		F	0	1	2			
Escape	PLINK	M	0	*1*	NA	${e}^{\beta_1}$	${e}^{\beta_1}$	${e}^{2{\beta}_1}$
		F	0	1	2			

^a^Expressions for calculating the OR are shown for the effect of one allele copy in men (OR_M_) and one or two copies in women (OR_W1_ and OR_W2_). For men, but not women, the OR is calculated differently based on whether the SNP is believed to be subject to XCI (and hence the coding scheme, PLINK or Clayton, that is chosen)

In this study, we employed two X chromosome-specific approaches that allow for modeling SNP effects depending on XCI status (inactivation vs. escape). In the first approach, we used biological data on which regions are likely to experience XCI to model SNP effects differently for regions subject to and escaping from XCI; this approach assumes that under a given coding scheme, the SNP effect is the same in males and females. Specifically, in regions believed to undergo XCI, we used the Clayton coding of male genotypes (0/2) and test the SNP effect while assuming that the minor allele in males has the same effect as two copies of the minor allele in females (OR_M_ = OR_W2_). On the other hand, in regions believed to escape XCI, we used the PLINK coding of male genotypes (0/1) and test the SNP effect while assuming that the minor allele in males has the same effect as one copy of the minor allele in females (OR_M_ = OR_W1_; Table 1). In the second approach, we fit a more flexible regression model that can model SNPs that are either subject to or escaping from XCI, without the need for prior biological knowledge of the XCI status. This approach also allows for SNP effects to differ in males and females. These approaches are compared in the context of investigating the genetics of BD-related traits.

### Approach 1: XCI-informed approach

#### Deriving a presumed XCI status for each X chromosome SNP

Previous work by Balaton et al. [31] derived a “consensus” inactivation status across multiple studies and multiple tissue types for approximately 400 genes on the X chromosome. To infer XCI status at a SNP level, we used the presumed XCI status for each gene (as given in “Additional file 1: Table S1.” from Balaton et al. [31]). Start and stop positions of all genes are per the transcription start and stop sites. Any SNPs overlapped by only “subject” genes (category: Subject) or only “escape” genes (categories: PAR and escape) were assigned the corresponding XCI status (“subject” or “escape”); SNPs lying between genes of the same type were also assigned the corresponding XCI status. SNPs between “subject” and “escape” genes or overlapping both “subject” and “escape” genes were assigned an XCI status of “unknown.”

#### Using an XCI status informed approach for testing associations between X chromosome SNPs and phenotype

To test association with each phenotype, a sex-adjusted logistic regression model (Eq. 1) was used:

1 $$ \mathrm{logit}\;\left(\mathrm{pheno}\right)={\upbeta}_0+{\upbeta}_1\;\left(\mathrm{SNP}\right)+{\upbeta}_2\;\left(\mathrm{sex}\right) $$

Sex was coded as 0 for females and 1 for males. Irrespective of presumed XCI status, the SNP variable in females was set equal to the number of copies of the minor allele. However, in males, the SNP variable’s coding depended on the presumed XCI status and hence the coding scheme chosen (Clayton or PLINK coding; Table 1). SNPs of unknown XCI status were modeled under both coding schemes (Clayton and PLINK), and Akaike information criterion (AIC) was used to determine which XCI status led to the better fitting model in each cohort (lower AIC indicates better model fit).

When XCI status at a SNP was unknown and the cohorts gave discordant presumed XCI statuses, the coding used for generating the cohort-specific summary statistics for the meta-analysis was the Clayton coding, since most of the X chromosome is subject to XCI.

### Approach 2: XCI-robust approach

In this second approach, a logistic regression model with a SNP-sex interaction term (Eq. 2) was employed, where the SNP variable was the count of copies of the minor allele, and a likelihood ratio test with two degrees-of-freedom (df) was used to jointly assess the significance of the SNP and SNP-sex interaction terms. Sex was coded as 1 for males and 0 for females.

2 $$ \mathrm{logit}\;\left(\mathrm{pheno}\right)={\upbeta}_0+{\upbeta}_1\;\left(\mathrm{SNP}\right)+{\upbeta}_2\;\left(\mathrm{sex}\right)+{\upbeta}_3\left(\mathrm{SNP}\right)\left(\mathrm{sex}\right) $$

To facilitate the interpretation of the top SNP effects in males and females, sex-stratified logistic regression analyses were conducted in Mayo and GAIN.

For all analyses, a chromosome-wide Bonferroni-corrected significance threshold was set by dividing 0.05 by the number of SNPs passing QC in the GAIN set prior to imputation (*P* = 0.05/26,662 = 1.88 × 10^−6^). Regression analyses were performed in R using the “glm” function. Analyses incorporated additional covariates for genetic ancestry as assessed by principal components, DIGS questionnaire version (for GAIN), and enrollment site (Mayo Clinic cohort only) when necessary. For the XCI-informed approach, meta-analysis of results from the Mayo and GAIN cohorts was conducted in METAL by weighting observations from each study inversely proportional to their standard errors [32]. For the XCI-robust approach, the *P* values from the 2df test in the Mayo and GAIN cohorts were combined by Fisher’s method to derive a joint *P* value implemented in R [29]. Meta-analyses of sex-stratified results from the Mayo and GAIN cohorts were performed using inverse-variance weighting using METAL [32] to estimate SNP effects in men and women separately for each phenotype.

### Candidate SNP study

Previously, Jancic et al. [33] analyzed the association of X chromosome SNPs with risk of suicide attempt in individuals with BD (983 suicide attempters, 1143 non-attempters), which included the individuals from the GAIN sample analyzed here. We attempted to replicate the top ten SNPs from that paper in the independent Mayo sample. The original work used the PLINK coding and sex-adjusted logistic regression to identify top SNPs. We applied the two X chromosome-specific approaches described here to the Mayo data. As all ten SNPs reported by Jancic lay in a region subject to XCI, the “XCI-informed approach” used Clayton coding for all of these SNPs.

### Annotation of lead SNPs

All lead SNPs reported in this paper were annotated to the nearest gene (not counting pseudo-genes or lncRNAs) using BioR [34] and Gr37Chp5 or by visual inspection in the University of California Santa Cruz (UCSC) Genome Browser. The GTEx database [35] was used to verify if any of the top SNPs are expression quantitative trait locus (eQTLs) in any tissue (FDR < 0.05) or are splice quantitative trait locus (sQTLs) (FDR < 0.05).

## Results

All characteristics of BD examined (RC, suicide attempt, BE, and AUD) were relatively common in both Mayo Clinic and GAIN datasets (Table 2). For both Mayo Clinic and GAIN, women were more likely than men to engage in BE or to have attempted suicide, and men were at greater risk for having an AUD. Additionally, RC was significantly more common for female cases (*P* = 0.004) for Mayo, although this was not true for GAIN (*P* = 0.580).

**Table 2:** Characteristics of bipolar disorder cases

	Mayo			GAIN		
	Male (*N* = 396)	Female (*N* = 573)	*P*	Male (*N* = 500)	Female (*N* = 501)	*P*
Age, mean (SD)	43.48 (15.78)	42.35 (14.82)	0.255	41.96 (13.01)	42.28 (12.86)	0.697
Rapid cycling, *N* (%)	211 (53.4)	357 (62.9)	0.004	159 (38.6)	175 (40.7)	0.580
Suicide attempt, *N* (%)	87 (22.1)	222 (39.2)	< 0.001	175 (36.0)	252 (50.9)	< 0.001
BE behavior, *N* (%)	61 (21.9)	131 (31.0)	0.011	30 (6.6)	94 (20.1)	< 0.001
Alcohol use disorder, *N* (%)	170 (44.4)	199 (35.9)	0.011	257 (51.4)	208 (41.5)	0.002

**Fig. 1:**
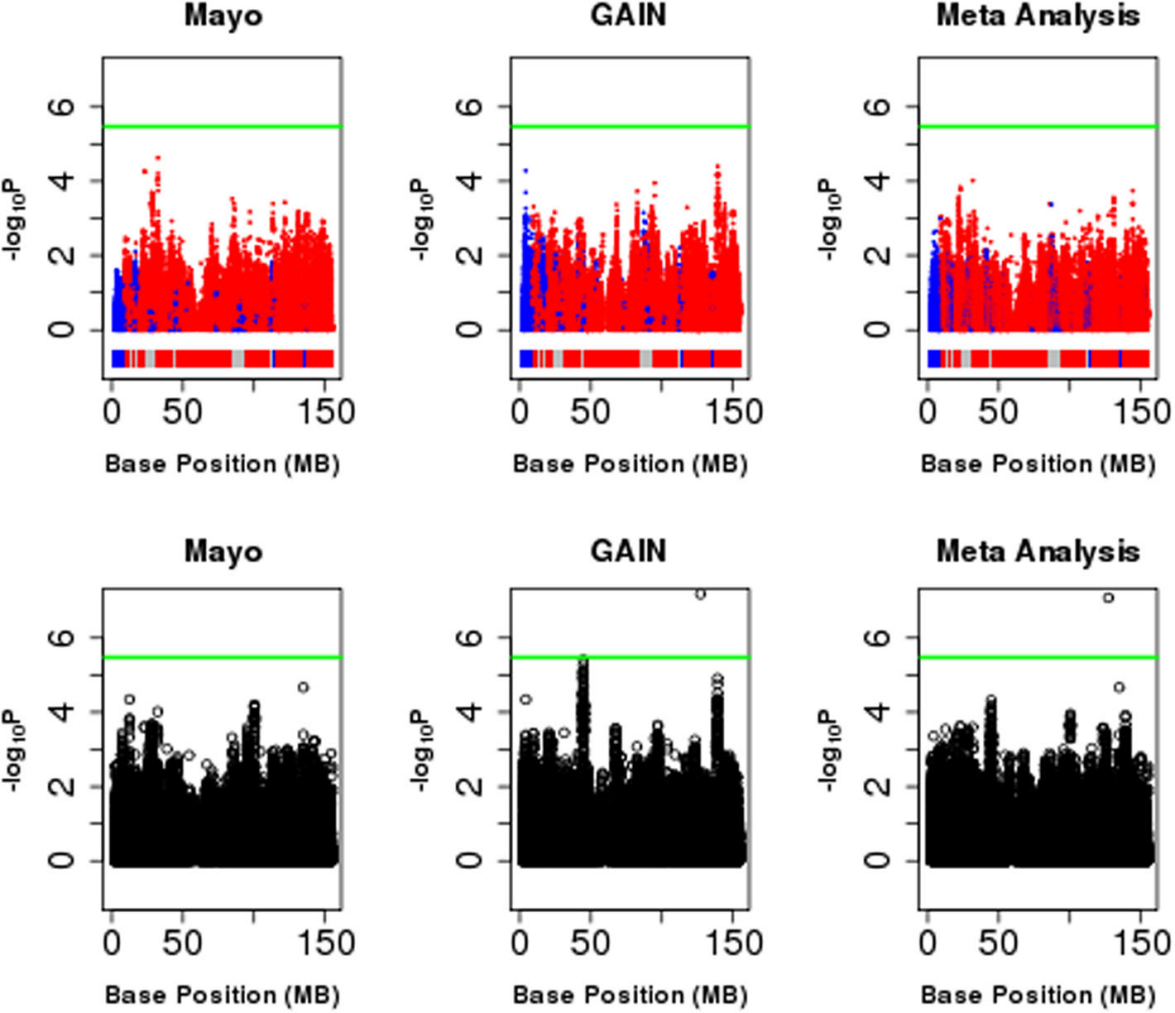
Association of X chromosome variants with BD. Top row denotes results from XCI-informed approach. Bottom row denotes results from XCI-robust approach. Green line denotes the study-wide significance threshold of 3.36 × 10^−6^. Domains as shown in the colored bars beneath the Manhattan plots for XCI-informed approach denote whether SNPs fall into regions experiencing (red) or escaping (blue) from X chromosome inactivation. Grey denotes regions for which a domain (subject or escaping) could not be assigned based on the paper by Balaton et al. [31]. SNPs are colored by the chosen XCI status used in the meta-analysis

X chromosome-wide results for all phenotypes under both the “XCI-informed approach” and the “XCI-robust approach” are displayed in Fig. 1 and Additional files 1, 2, 3, and 4. Using the “XCI-informed approach,” which examines marginal SNP effects, no SNPs were identified that were significantly associated with BD or any of its sex-specific symptoms and comorbidities (Additional file 5: Table S1). However, using the “XCI-robust approach,” which considers SNP-sex interactions, the SNP rs5932307 was significantly associated with BD (*P* = 8.31E−8; Table 3). The minor A allele was associated with higher odds of BD, with a stronger effect in females (OR_W2_ = 3.86 vs. OR_M_ = 1.13). This SNP is downstream of the ACTRT1 gene, which has the highest gene expression in the testes [35] and encodes a beta actin-like protein that is suggested to be important for spermatid formation [36]. It has not been identified as an expression quantitative trait locus (eQTL) in any tissues or spliceQTL. However, we should note that this SNP marginally deviates from Hardy-Weinberg Equilibrium in female controls in the GAIN sample (*P* = 1.2E−4), but not in the Mayo sample (*P* > 0.05).

**Table 3:** Top SNPs under “XCI-robust” approach

						XCI-informed					XCI-robust			
Phenotype	SNP	Alleles (min/maj)	Nearest gene	Cohort	MAF	Chosen XCI status	OR_M_	OR_W1_	OR_W2_	*P*	OR_M_	OR_W1_	OR_W2_	*P*
Case status	rs5932307	A/G	ACTRT1	Mayo	0.08	S	1.59 (1.04–2.43)	1.26 (1.02–1.56)	1.59 (1.04–2.43)	0.03	1.39 (0.82–2.36)	1.42 (1.00–2.02)	2.02 (1.00–4.08)	0.06
				GAIN	0.07	S	1.60 (1.12–2.30)	1.27 (1.06–1.52)	1.60 (1.12–2.30)	0.01	1.01 (0.67–1.50)	3.54 (2.20–5.69)	12.52 (4.85–32.34)	6.5E−08
				*Meta*		*S*	*1.60 (1.21–2.10)*	*1.26 (1.10–1.45)*	*1.60 (1.21–2.10)*	*8.2E−04*	*1.13 (0.82–1.56)*	*1.96 (1.48−2.60)*	*3.86 (2.19–6.78)*	*8.3E−08*
Rapid cycling	rs5933727	G/C	TBL1X	Mayo	0.07	E*	1.91 (1.19–3.06)	1.91 (1.19–3.06)	3.65 (1.42–9.38)	0.01	1.31 (0.56–3.07)	2.25 (1.26–4.02)	5.08 (1.60–16.18)	0.01
				GAIN	0.08	E*	0.38 (0.23–0.62)	0.38 (0.23–0.62)	0.15 (0.05–0.39)	1.2E−04	0.76 (0.34–1.71)	0.27 (0.14–0.51)	0.07 (0.02–0.26)	3.1E−05
				*Meta*		E*	*0.77 (0.55–1.08)*	*0.77 (0.55–1.08)*	*0.60 (0.30–1.18)*	*0.14*	*0.99 (0.55–1.77)*	*0.86 (0.56–1.31)*	*0.73 (0.31–1.73)*	*6.2E−06*
Suicide attempt	rs5975146	A/G	XPNPEP2	Mayo	0.12	S	0.47 (0.25–0.85)	0.68 (0.50–0.92)	0.47 (0.25–0.85)	0.01	0.10 (0.02–0.45)	0.99 (0.67–1.46)	0.98 (0.44–2.15)	6.2E−04
				GAIN	0.11	S	1.77 (1.09–2.87)	1.33 (1.04–1.69)	1.77 (1.09–2.87)	0.02	1.07 (0.58–1.99)	2.15 (1.39–3.32)	4.63 (1.94–11.05)	1.7E−03
				*Meta*		*S*	*1.05 (0.72–1.53)*	*1.02 (0.85–1.24)*	*1.05 (0.72–1.53)*	*0.80*	*0.76 (0.43–1.33)*	*1.40 (1.05–1.88)*	*1.97 (1.10–3.53)*	*1.5E−05*
Binge eating	rs6627188	A/C	CNGA2	Mayo	0.14	S	0.17 (0.08–0.39)	0.41 (0.28–0.62)	0.17 (0.08–0.39)	2.0E−05	0.06 (0.01–0.50)	0.48 (0.30–0.78)	0.23 (0.09–0.61)	4.0E−06
				GAIN	0.13	S	1.65 (0.86–3.14)	1.28 (0.93–1.77)	1.65 (0.86–3.14)	0.13	2.00 (0.76–5.27)	1.21 (0.78–1.86)	1.45 (0.61–3.46)	0.31
				*Meta*		*S*	*0.68 (0.41–1.13)*	*0.83 (0.64–1.06)*	*0.68 (0.41–1.13)*	*0.14*	*1.08 (0.45–2.60)*	*0.80 (0.58–1.11)*	*0.64 (0.34–1.23)*	*1.8E−05*
Alcohol use disorder	rs145649722	G/C	CLCN5	Mayo	0.04	S	0.78 (0.28–2.15)	0.88 (0.53–1.47)	0.78 (0.28–2.15)	0.63	0.73 (0.16–3.29)	0.92 (0.46–1.83)	0.84 (0.21–3.36)	0.88
				GAIN	0.05	S	2.60 (1.24–5.47)	1.61 (1.11–2.34)	2.60 (1.24–5.47)	0.01	8.29 (2.50–27.51)	0.58 (0.29–1.18)	0.34 (0.08–1.39)	4.1E−05
				*Meta*		*S*	*1.68 (0.92–3.05)*	*1.30 (0.96–1.75)*	*1.68 (0.92–3.05)*	*0.09*	*3.20 (1.26–8.15)*	*0.74 (0.45–1.21)*	*0.55 (0.21–1.47)*	*4.1E−04*

Odds ratios associated with an increase of one minor allele copy in men (OR_M_) or women (OR_W1_) or an increase of two copies in women (OR_W2_) are reported for two different analysis approaches. The XCI-informed approach employed a sex-adjusted logistic regression model (Eq. 1), but coded the SNP variable differently dependent on presumed XCI status (listed in the “Chosen XCI status” column). SNPs were assigned a status of subject (S) or escaping from inactivation, based on prior work on which regions of the X chromosome experience inactivation. For SNPs in regions of unknown XCI status (entries with asterisk in the “Chosen XCI status” column), presumed XCI status was determined by fitting the model using both the PLINK and Clayton coding schemes and using Akaike information criterion to select the more appropriate model. The XCI-robust approach employed a sex-adjusted logistic regression model with a SNP-sex interaction term (Eq. 2). The significance of the SNP and SNP-sex terms in the model was assessed by a *χ*^2^ test with two degrees-of-freedom (2df)

Italics denote the results of the meta-analysis

Top SNPs for suicide attempt and AUD under the “XCI-robust approach,” although not significant after Bonferroni correction, were single-tissue eQTLs (Table 3). The SNP most strongly associated with suicide attempt was rs5975146, an eQTL of the gene X-prolyl aminopeptidase 2 (XPNPEP2) in both tibial nerve and adipose tissue. The meta-analysis of results from Mayo and GAIN under the “XCI-robust approach,” which allows SNP effects to differ by sex, suggests that the minor A allele of rs5975146 may be associated with greater risk of suicide attempt, but only among females (OR_W1_ = 1.40, *P*_2df_ = 1.5E−5). Additionally, the SNP most associated with AUD (rs145649722) was an eQTL of CLCN5 in the skin. The results from the meta-analysis suggest that the minor G allele of rs145649722 may be associated with greater odds of AUD, primarily in males (OR_M_ = 3.20, OR_W2_ = 0.55, *P*_2df_ = 4.1E−4).

We analyzed ten SNPs most strongly associated with suicide attempt in prior work [33] in the Mayo Clinic cohort. None of these SNPs was even nominally associated (*P* < 0.05) with the risk of suicide attempt in the independent Mayo Clinic sample (Additional file 6: Table S2). When the GAIN data was analyzed using the XCI-informed and XCI-robust methods, only two SNPs were nominally associated (rs5909133, *P*_informed_ = 0.0037, *P*_robust_ = 0.014; rs695214, *P*_informed_ = 0.00052, *P*_robust_ = 0.0013); this cannot be considered an independent replication, as the prior study included the GAIN data.

## Discussion

In this study, we examined the association of X chromosome SNPs with sex-associated characteristics of BD using two different X chromosome-specific analysis approaches. These approaches consider the sex-specific nature of the X chromosome and the process of XCI and allow for a more flexible interpretation of the findings.

The sex associations of the BD characteristics are as expected based on prior work, including higher rates of RC, lifetime history of suicide attempt, and greater prevalence of BE in women, as well as greater prevalence of AUDs in men.

The SNP rs5932307 was significantly associated with BD under the “XCI-robust approach” (*P* = 8.3 × 10^−8^), even with a conservative, Bonferroni-corrected significance threshold of *P* = 1.88 × 10^−6^. This contrasts with results from a recent GWAS that employed a two-stage methodology with independent discovery (7467 cases/27,303 controls) and replication samples (2313 cases/3489 controls); in that study, despite the larger sample size of the discovery cohort, no X chromosome SNPs passed the threshold (*P* = 1 × 10^−6^) to advance to testing in the replication sample [1]. However, this may be because different approaches to association testing were employed. In the previous study, the association test used the Clayton coding, which assumes that the minor allele in males has the same effect as two copies of the minor allele in females. However, the approach that yielded the significant result for our analysis was the “XCI-robust approach,” which allowed the effect of the SNP to differ by sex. The potential importance of allowing SNP effects to differ by sex is highlighted by the fact that for this SNP, sex-stratified analyses suggest that the minor allele is more strongly associated with BD for females (OR_W2_ = 3.86, 95% CI 2.19–6.78) than for males (OR_M_ = 1.13, 95% CI 0.82–1.56). However, this result should be interpreted cautiously given that this SNP showed some deviation from Hardy Weinberg equilibrium in one of the analyzed datasets.

Although not significant after multiple testing correction, the SNP most strongly associated with suicide attempt (rs5975146) was an eQTL of the X-prolyl aminopeptidase 2 (XPNPEP2) in both tibial nerve and adipose tissue, and the SNP most associated with AUD (rs145649722) was an eQTL of CLCN5 in the skin. The gene CLCN5 encodes the protein chloride channel 5 (Clc-5), and one study found the gene CLCN5 to be differentially methylated in brain tissue from obsessive-compulsive disorder subjects and controls [34].

Candidate SNPs most significantly associated with risk of suicide attempt in a prior study in a BD population of which the GAIN data was a subset [33] were not significantly associated with suicide attempt within our Mayo cohort, regardless of coding or approach, with most OR estimates close to one. This may have been due to differences in methodology, as most of these SNPs were also not associated in our analysis of the GAIN data, with the exception of rs695214.

Importantly, correct interpretation of X chromosome association results depends on the statistical model that was fit and genotype coding that was used, which reflect assumptions that were made. When interpreting effect size for X chromosome SNPs, multiple ORs are informative. Whereas for autosomes ORs are commonly reported for the change of one allele copy (assuming an additive model for allele effects), it is less clear what is most appropriate to report for X chromosome variants, because the effect of the SNP varies with sex. Under the “XCI-informed approach,” for SNPs lying in regions that escape from XCI, the assumption is that the OR in males (OR_M_) is the same as females for a change of one allele copy (OR_W1_). However, for SNPs lying in regions experiencing XCI, the effect of a change of one allele copy in males (OR_M_) is expected to be comparable to a change of two copies in females (OR_W2_). These assumptions are implicit in the “XCI-informed approach,” which assumes a log-additive effect of SNP in females.

While the “XCI-robust approach” that includes SNP-sex interactions also assumes that the effects of SNPs are log-additive in females, it is more flexible because the effect of a SNP can vary by sex. OR_M_ is not constrained to equal the effect of the SNP in females (OR_W1_ or OR_W2_), which even allows for a SNP to exhibit a protective effect in one sex and to be a risk factor for the other sex. It is worth noting that the “XCI-informed approach” and the “XCI-robust approach” are designed to detect different genetic effects on the phenotype. The “XCI-informed approach” examines the main effect of the SNP variable on the phenotype, whereas the “XCI-robust approach” with the 2df test reflects the joint importance of the SNP and SNP-sex interaction terms, and hence is sensitive not only to the main effects but also to differences in the SNP effect between sexes.

The importance of allowing for this flexibility in the model can be seen by looking at top SNPs for each phenotype under the more restrictive “XCI-informed approach.” All of these SNPs are in a region subject to XCI, which would lead one to predict those SNPs have the same effect for one allele in males as two copies in females (i.e., OR_M_ = OR_W2_). However, examining the sex-stratified ORs for those SNPs (Additional file 5: Table S1) shows that many of those SNPs potentially have SNP effects that do not follow the expected theoretical pattern. For example, the top SNP for AUD under the “XCI-informed approach,” rs62587381, has an estimated OR in males that is much greater than in females (OR_M_ = 4.32 versus OR_W2_ = 1.85).

One might be concerned that the increased flexibility of the model might come at the expense of reduced power to detect genetic differences. However, this does not appear to be a major concern, at least in our study. For three of the five top SNPs for each phenotype under the “XCI-informed approach,” we observed a *P* value within an order of magnitude for the “XCI-robust approach.” Additionally, only the “XCI-robust approach” resulted in a significant finding for any of the phenotypes studied. However, a disadvantage of the “XCI-robust approach” when used across datasets that are subsequently meta-analyzed is that it relies on a two degrees-of freedom likelihood ratio test statistic that does not retain the directionality of the SNP effect, which can lead to difficulties in interpreting meta-analysis results.

Selection of prior gene-level XCI states is necessary for the “XCI-informed approach.” We used the XCI consensus states described in Balaton et al. [31], because they were assessed across multiple studies and multiple tissue types and could be considered generally applicable, and it is unclear which tissue type might best inform BD risk. Because XCI patterns are known to be tissue-specific, a tissue-specific XCI source could be used for conditions with clearly defined normal tissue types, if it exists [13]. Failing to properly account for tissue-specific patterns could possibly lead to a reduction in power for the “XCI-informed approach” if the wrong XCI state is modeled. An advantage of the “XCI-robust approach” is that it does not rely on specification of the tissue-specific XCI pattern. Furthermore, the “XCI-robust approach” can also accommodate the phenomenon of partial or incomplete escape from XCI, which is not accounted for in the “XCI-informed approach.”

Neither the “XCI-informed” or the “XCI-robust” approaches directly account for genes that are homologous across the X and Y chromosome (gametologs), as they do not incorporate Y chromosome data from males. The “XCI-informed” approach treats SNPs within these genes as escaping from XCI, whereas the “XCI-robust” approach does not make any assumptions about XCI status. This suggests that development of methods that incorporate X and Y data for studying these regions would be valuable.

Strengths of our work include the investigation of the role of X chromosome genetic variants to multiple symptoms and comorbidities of BD with known sex-differences in prevalence, and the use of two methods of analysis that can model the effect of SNPs both subject to and escaping from XCI. Importantly, we developed a new approach for analyzing X chromosome genetic variants that incorporates prior biological information on XCI status. However, our study also has limitations. The biological relevance of our observed associations is unclear, and laboratory validation required to establish biological associations is beyond the scope of this work, as is a comparison of the genetic versus hormonal influences on sex differences in BD. The relatively small sample size limited statistical power and makes interpretation of the significance of our findings difficult. Additionally, our cohorts were composed solely of individuals of European ancestry. Future work in more ethnically diverse cohorts or larger cohorts such as the Psychiatric Genomics Consortium might allow us to discover new X chromosome genetic variants that are important to BD risk and allow for findings with greater generalizability.

This work provides a basis for future methodological studies. Future work should extend both approaches to incorporate data from the Y chromosome in males for the XY gametolog genes. The relative merits of the two approaches should be more rigorously assessed by simulation studies assessing type I error and statistical power, as well as comparison to other existing approaches [37]. Alternate approaches could be explored, such as prioritizing SNPs in sex-biased genes or using Bayesian methods or model averaging [38], which could reflect the uncertainty that exists about a locus’ XCI status. In addition, statistical approaches to determine the likely genetic architecture by which genotypes alter phenotypes (e.g., additivity vs. dominance of allelic effects) could also be pursued; additionally, information about the genetic architecture may also imply XCI status. Finally, the versatility and relative ease of implementation of our approach should encourage its broad application, particularly in conditions where X chromosome involvement is suggested, but few if any specific genes have been identified.

## Perspectives and significance

In conclusion, we employed two different approaches to the analysis of X chromosome genetic variants that are able to model SNPs both subject to and escaping from XCI. In the “XCI-informed approach,” we used biological information regarding what regions of the X chromosome undergo XCI to code the SNP variable differently for regions believed to undergo versus escape from inactivation. In the “XCI-robust approach,” a more flexible model with a SNP-sex interaction term was fit that allowed for SNPs both in regions of inactivation and escape, without the need for prior knowledge as to the true XCI status. We also describe how the SNP effect sizes can be interpreted for each sex based on the model that was fit.

Neither approach identified SNPs that were significantly associated with sex-specific symptoms of BD, although the interaction approach identified a SNP (rs5932307) associated with risk of BD (*P* = 8.31 × 10^−8^). Future work in larger, independent cohorts is needed to replicate this finding, but our work highlights the importance of applying X chromosome-specific methods and careful interpretation of the results when analyzing phenotypes with known sex differences.

## Supplementary information

**Additional file 1: Figure S1.** Association of X chromosome genetic variants with RC. Top row denotes results from XCI-Informed Approach. Bottom row denotes results from XCI-Robust Approach. Green line denotes the study wide significance threshold of 3.36x10^-6^. Domains as shown in the colored bars beneath the Manhattan plots for XCI-Informed Approach denote whether SNPs fall into regions experiencing (red) or escaping (blue) from X chromosome inactivation. Grey denotes regions for which a domain (subject or escaping) could not be assigned based on the paper by Balaton et al [31]. SNPs are colored by the chosen XCI status used in the meta-analysis.

**Additional file 2: Figure S2.** Association of X chromosome genetic variants with attempted suicide. Top row denotes results from XCI-Informed Approach. Bottom row denotes results from XCI-Robust Approach. Green line denotes the study wide significance threshold of 3.36x10^-6^. Domains as shown in the colored bars beneath the Manhattan plots for XCI-Informed Approach denote whether SNPs fall into regions experiencing (red) or escaping (blue) from X chromosome inactivation. Grey denotes regions for which a domain (subject or escaping) could not be assigned based on the paper by Balaton et al [31]. SNPs are colored by the chosen XCI status used in the meta-analysis.

**Additional file 3: Figure S3.** Association of X chromosome genetic variants with BE. Top row denotes results from XCI-Informed Approach. Bottom row denotes results from XCI-Robust Approach. Green line denotes the study wide significance threshold of 3.36x10^-6^. Domains as shown in the colored bars beneath the Manhattan plots for XCI-Informed Approach denote whether SNPs fall into regions experiencing (red) or escaping (blue) from X chromosome inactivation. Grey denotes regions for which a domain (subject or escaping) could not be assigned based on the paper by Balaton et al [31]. SNPs are colored by the chosen XCI status used in the meta-analysis.

**Additional file 4: Figure S4.** Association of X Chromosome Genetic Variants with AUD. Top row denotes results from XCI-Informed Approach. Bottom row denotes results from XCI-Robust Approach. Green line denotes the study wide significance threshold of 3.36x10^-6^. Domains as shown in the colored bars beneath the Manhattan plots for XCI-Informed Approach denote whether SNPs fall into regions experiencing (red) or escaping (blue) from X chromosome inactivation. Grey denotes regions for which a domain (subject or escaping) could not be assigned based on the paper by Balaton et al [31]. SNPs are colored by the chosen XCI status used in the meta-analysis.

**Additional file 5: Table S1.** Top SNPs under “XCI-informed” Approach.

**Additional file 6: Table S2.** Candidate SNPs for Association with Suicide Attempt.

## Data Availability

The datasets generated and/or analyzed for the GAIN cohort during the current study are available and were collected in previous work [17] and deposited in the dbGaP repository [26] (accession #: phs000017.v3.p1). Datasets generated and/or analyzed for the Mayo cohort contain protected health information and will not be shared to protect patient privacy

## Abbreviations

AIC: Akaike information criterion
AUD: Alcohol use disorder
BD: Bipolar disorder
BE: Binge eating behavior
df: Degrees-of-freedom
DIGS: Diagnostic Interview of Genetic Studies
eQTL: Expression quantitative trait locus
GAIN: Genetic Association Information Network
MAF: Minor allele frequency
OR: Odds ratio
OR_M_: Odds ratio in males for a change of 1 allele copy
OR_W1_: Odds ratio in females for a change of 1 allele copy
OR_W2_: Odds ratio in females for a change of 2 allele copies
PAR: Pseudoautosomal region
QC: Quality control
QTL: Quantitative trait locus
RC: Rapid cycling
SNP: Single nucleotide polymorphism
