# Supplementary figures and images for: Statistical methods for testing X chromosome variant associations: application to sex-specific characteristics of bipolar disorder
Source: Biol Sex Differ. 2019 Dec 9;10:57. doi: 10.1186/s13293-019-0272-4 (PMC6902568; doi:10.1186/s13293-019-0272-4)

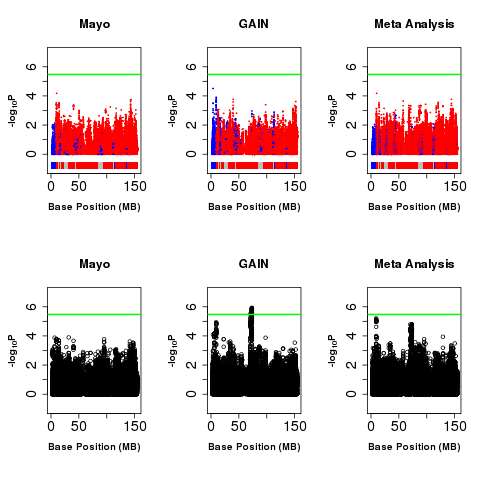

Supplement: Supplementary file 1 — Additional file 1: Figure S1. Association of X chromosome genetic variants with RC. Top row denotes results from XCI-Informed Approach. Bottom row denotes results from XCI-Robust Approach. Green line denotes the study wide significance threshold of 3.36x10^-6^. Domains as shown in the colored bars beneath the Manhattan plots for XCI-Informed Approach denote whether SNPs fall into regions experiencing (red) or escaping (blue) from X chromosome inactivation. Grey denotes regions for which a domain (subject or escaping) could not be assigned based on the paper by Balaton et al [31]. SNPs are colored by the chosen XCI status used in the meta-analysis. [file 13293_2019_272_MOESM1_ESM.tiff]

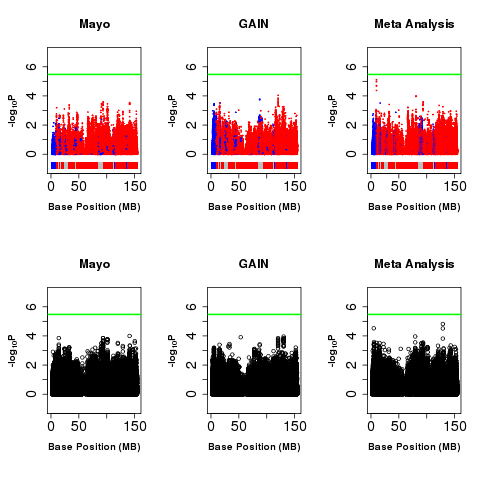

Supplement: Supplementary file 2 — Additional file 2: Figure S2. Association of X chromosome genetic variants with attempted suicide. Top row denotes results from XCI-Informed Approach. Bottom row denotes results from XCI-Robust Approach. Green line denotes the study wide significance threshold of 3.36x10^-6^. Domains as shown in the colored bars beneath the Manhattan plots for XCI-Informed Approach denote whether SNPs fall into regions experiencing (red) or escaping (blue) from X chromosome inactivation. Grey denotes regions for which a domain (subject or escaping) could not be assigned based on the paper by Balaton et al [31]. SNPs are colored by the chosen XCI status used in the meta-analysis. [file 13293_2019_272_MOESM2_ESM.tiff]

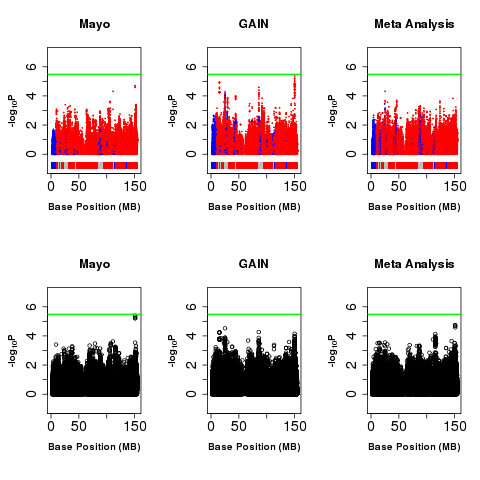

Supplement: Supplementary file 3 — Additional file 3: Figure S3. Association of X chromosome genetic variants with BE. Top row denotes results from XCI-Informed Approach. Bottom row denotes results from XCI-Robust Approach. Green line denotes the study wide significance threshold of 3.36x10^-6^. Domains as shown in the colored bars beneath the Manhattan plots for XCI-Informed Approach denote whether SNPs fall into regions experiencing (red) or escaping (blue) from X chromosome inactivation. Grey denotes regions for which a domain (subject or escaping) could not be assigned based on the paper by Balaton et al [31]. SNPs are colored by the chosen XCI status used in the meta-analysis. [file 13293_2019_272_MOESM3_ESM.tiff]

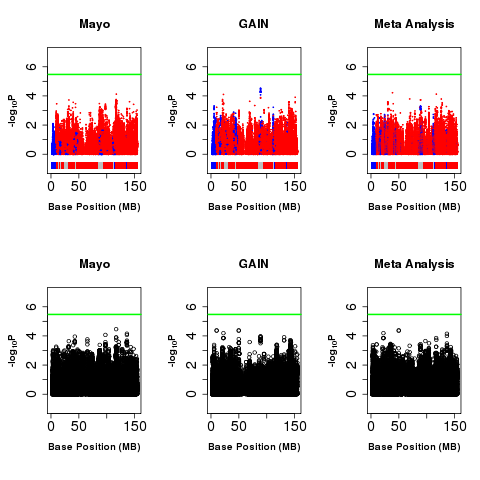

Supplement: Supplementary file 4 — Additional file 4: Figure S4. Association of X Chromosome Genetic Variants with AUD. Top row denotes results from XCI-Informed Approach. Bottom row denotes results from XCI-Robust Approach. Green line denotes the study wide significance threshold of 3.36x10^-6^. Domains as shown in the colored bars beneath the Manhattan plots for XCI-Informed Approach denote whether SNPs fall into regions experiencing (red) or escaping (blue) from X chromosome inactivation. Grey denotes regions for which a domain (subject or escaping) could not be assigned based on the paper by Balaton et al [31]. SNPs are colored by the chosen XCI status used in the meta-analysis. [file 13293_2019_272_MOESM4_ESM.tiff]
