# Supplementary material for: Statistical methods for testing X chromosome variant associations: application to sex-specific characteristics of bipolar disorder
Source: Biol Sex Differ. 2019 Dec 9;10:57. doi: 10.1186/s13293-019-0272-4 (PMC6902568; doi:10.1186/s13293-019-0272-4)
Supplement: Supplementary file 5 — Additional file 5: Table S1. Top SNPs under “XCI-informed” Approach. [file 13293_2019_272_MOESM5_ESM.docx]

**Supplementary Table 1.** Top SNPs under “XCI-informed” Approach.

|  |  |  |  |  |  | **XCI-informed** | | | | | **XCI-robust** | | | |
| --- | --- | --- | --- | --- | --- | --- | --- | --- | --- | --- | --- | --- | --- | --- |
| **Pheno-**  **type** | **SNP** | **Alleles (Min/Maj)** | **Nearest Gene** | **Cohort** | **MAF** | **Chosen XCI Status** | **OR_M_** | **OR_W1_** | **OR_W2_** | **P** | **OR_M_** | **OR_W1_** | **OR_W2_** | **P.2df** |
| case status | rs140988523 | C/T | DMD | Mayo | 0.15 | S | 2.04  (1.43-2.92) | 1.43  (1.19-1.71) | 2.04  (1.43-2.92) | 9.63E-05 | 2.30  (1.46-3.62) | 1.27  (0.95-1.72) | 1.62  (0.89-2.95) | 2.48E-04 |
|  |  |  |  | GAIN | 0.16 | SNP Fails QC within Cohort | | | | | | | | |
|  |  |  |  | **Meta** |  | **S** | **2.04**  **(1.43-2.92)** | **1.43**  **(1.19-1.71)** | **2.04**  **(1.43-2.92)** | **9.55E-05** | **2.30(1.46-3.62)** | **1.27(0.95-1.72)** | **1.62(0.89-2.95)** | **2.48E-04** |
| rapid cycling | rs1013536 | A/C | WWC3 | Mayo | 0.39 | S | 0.51(0.37-0.71) | 0.71(0.60-0.84) | 0.51(0.37-0.71) | 6.64E-05 | 0.43(0.28-0.67) | 0.80(0.62-1.04) | 0.65(0.38-1.09) | 1.61E-04 |
|  |  |  |  | GAIN | 0.39 | SNP Fails QC within Cohort | | | | | | | | |
|  |  |  |  | **Meta** |  | **S** | **0.51**  **(0.37-0.71)** | **0.71**  **(0.60-0.84)** | **0.51**  **(0.37-0.71)** | **6.59E-05** | **0.43(0.28-0.67)** | **0.80(0.62-1.04)** | **0.65(0.38-1.09)** | **1.61E-04** |
| suicide attempt | rs5933755 | T/C | GPR143 | Mayo | 0.16 | S* | 2.00  (1.26-3.16) | 1.41  (1.12-1.78) | 2.00  (1.26-3.16) | 3.05E-03 | 1.72(0.92-3.20) | 1.55(1.09-2.20) | 2.40(1.19-4.84) | 9.84E-03 |
|  |  |  |  | GAIN | 0.16 | S* | 2.01(1.34-3.02) | 1.42(1.16-1.74) | 2.01(1.34-3.02) | 8.04E-04 | 1.80(1.10-2.93) | 1.61(1.11-2.35) | 2.61(1.23-5.51) | 2.64E-03 |
|  |  |  |  | **Meta** |  | **S*** | **2.00**  **(1.48-2.72)** | **1.42**  **(1.22-1.65)** | **2.00**  **(1.48-2.72)** | **7.78E-06** | **1.76(1.20-2.59)** | **1.58(1.22-2.04)** | **2.49(1.49-4.16)** | **3.00E-04** |
| binge eating | rs112485515 | C/A | ARX | Mayo | 0.15 | S | 2.38  (1.34-4.23) | 1.54  (1.16-2.06) | 2.38  (1.34-4.23) | 3.09E-03 | 3.06  (1.40-6.68) | 1.34  (0.87-2.05) | 1.79  (0.76-4.20) | 0.01 |
|  |  |  |  | GAIN | 0.15 | S | 2.36  (1.29-4.31) | 1.54  (1.14-2.07) | 2.36  (1.29-4.31) | 5.27E-03 | 2.62  (1.15-5.97) | 1.37  (0.87-2.16) | 1.87  (0.75-4.67) | 0.02 |
|  |  |  |  | **Meta** |  | **S** | **2.37(1.56-3.59)** | **1.54**  **(1.25-1.89)** | **2.37**  **(1.56-3.59)** | **4.74E-05** | **2.84**  **(1.61-5.01)** | **1.35**  **(0.99-1.85)** | **1.83**  **(0.98-3.41)** | **2.26E-03** |
| alcohol use disorder | rs62587381 | A/G | TSPAN7 | Mayo | 0.06 | S | 4.07  (1.81-9.12) | 2.02  (1.35-3.02) | 4.07  (1.81-9.12) | 6.64E-04 | 6.13  (1.84-20.43) | 1.54  (0.84-2.81) | 2.37  (0.71-7.88) | 1.10E-03 |
|  |  |  |  | GAIN | 0.05 | S | 2.46  (1.14-5.32) | 1.57  (1.07-2.31) | 2.46  (1.14-5.32) | 0.02 | 3.41  (1.27-9.16) | 1.17  (0.60-2.28) | 1.36  (0.36-5.20) | 0.03 |
|  |  |  |  | **Meta** |  | **S** | **3.13**  **(1.79-5.46)** | **1.77**  **(1.34-2.34)** | **3.13**  **(1.79-5.46)** | **6.09E-05** | **4.32**  **(2.01-9.27)** | **1.36**  **(0.87-2.13)** | **1.85**  **(0.76-4.53)** | **4.25E-04** |

Odds ratios associated with an increase of one minor allele copy in men (OR_M_) or women (OR_W1_) or an increase of two copies in women (OR_W2_) are reported for two different analysis approaches. The XCI-informed approach employed a sex-adjusted logistic regression model (Equation 1), but coded the SNP variable differently dependent on presumed XCI status (listed in ‘Chosen XCI Status’ column). SNPs were assigned a status of subject (S) or escaping from inactivation, based on prior work on which regions of the X chromosome experience inactivation. For SNPs in regions of unknown XCI status (starred entries in the ‘Chosen XCI status’ column), presumed XCI status was determined by fitting the model using both the PLINK and Clayton coding schemes and using Akaike information criterion to select the more appropriate model. The XCI-robust approach employed a sex-adjusted logistic regression model with a SNP-sex interaction term (Equation 2). The significance of the SNP and SNP-sex terms in the model was assessed by a χ^2^ test with 2 degrees-of-freedom (2df).
