# Supplementary material for: Statistical methods for testing X chromosome variant associations: application to sex-specific characteristics of bipolar disorder
Source: Biol Sex Differ. 2019 Dec 9;10:57. doi: 10.1186/s13293-019-0272-4 (PMC6902568; doi:10.1186/s13293-019-0272-4)
Supplement: Supplementary file 6 — Additional file 6: Table S2. Candidate SNPs for Association with Suicide Attempt. [file 13293_2019_272_MOESM6_ESM.docx]

**Supplementary Table 2.** Candidate SNPs for Association with Suicide Attempt.

|  |  |  |  | **Jancic** | | **XCI-informed** | | | **XCI-robust** |
| --- | --- | --- | --- | --- | --- | --- | --- | --- | --- |
| **SNP** | **Alleles (Min/Maj)^a^** | **Nearest Gene** | MAF.Mayo | **OR** | **P** | **OR_M_ =OR_W2_** | **OR_W1_** | **P** | **P.2df** |
| rs1002116 | G/A | NXT2 | 0.38 | 1.29 | 4.05E-04 | 0.97(0.68-1.40) | 0.99(0.82-1.18) | 0.88 | 0.56 |
| rs12555996 | G/T | KCNE5 | 0.38 | 1.3 | 2.86E-04 | 0.95(0.66-1.37) | 0.98(0.81-1.17) | 0.79 | 0.47 |
| rs1547612 | A/G | NXT2 | 0.38 | 1.29 | 4.05E-04 | 0.97(0.68-1.40) | 0.99(0.82-1.18) | 0.88 | 0.56 |
| rs2347955 | A/G | NXT2 | 0.38 | 1.29 | 3.40E-04 | 0.96(0.67-1.38) | 0.98(0.82-1.18) | 0.83 | 0.51 |
| rs5909133 | G/A | SH3KBP1 | 0.09 | 1.6 | 1.07E-04 | 1.12(0.62-2.00) | 1.06(0.79-1.41) | 0.71 | 0.13 |
| rs695214 | A/G | GRIA3 | 0.08 | 1.58 | 4.01E-04 | 0.91(0.49-1.67) | 0.95(0.70-1.29) | 0.76 | 0.07 |
| rs839370 | G/A | KCNE5 | 0.38 | 1.29 | 3.48E-04 | 0.95(0.66-1.37) | 0.98(0.81-1.17) | 0.79 | 0.47 |
| rs839382 | C/G | KCNE5 | 0.37 | 1.31 | 1.29E-04 | 0.94(0.65-1.36) | 0.97(0.81-1.17) | 0.74 | 0.50 |
| rs844441 | T/C | KCNE5 | 0.37 | 1.31 | 1.66E-04 | 0.96(0.66-1.39) | 0.98(0.81-1.18) | 0.82 | 0.55 |
| rs942602 | G/A | NXT2 | 0.38 | 1.29 | 4.05E-04 | 0.94(0.65-1.36) | 0.97(0.81-1.17) | 0.75 | 0.50 |

a Minor allele reported is the minor allele within the Mayo cohort

The 10 SNPs most strongly associated with risk of suicide attempt in a previous sample were analyzed in our independent, Mayo sample. The odds-ratios reported in Jancic et al reflects odds associated with one copy of the minor and were calculated using a sex-adjusted logistic regression model with the PLINK coding. All SNPs fell in a region classified by Balaton et al. as experiencing XCI, so the XCI-informed approach used the Clayton coding for all SNPs. The 2df test for the XCI-robust approach jointly assesses the important of the SNP and SNP-sex interactions terms in a sex-adjusted logistic regression model. All odds-ratios reported here are in reference to the change of one (OR_M_, OR_W1_) or two (OR_W2_) copies of the minor allele. The “MAF” column reports the minor allele frequency within the Mayo cohort.
